# Rigid Bronchoscopy in Foreign Body Aspiration Diagnosis and Treatment in Children

**DOI:** 10.3390/children8121206

**Published:** 2021-12-20

**Authors:** Aleksandra Pietraś, Marcin Markiewicz, Grażyna Mielnik-Niedzielska

**Affiliations:** Department of Pediatric Otolaryngology, Phoniatrics and Audiology, Medical University of Lublin, 20-093 Lublin, Poland; markiewiczmar@gmail.com (M.M.); grazyna.mielnik-niedzielska@umlub.pl (G.M.-N.)

**Keywords:** airway obstruction, bronchoscopy, child health, foreign bodies, respiratory aspiration

## Abstract

Foreign body aspiration in children is a common condition and can bring about serious undesired results. Fast and accurate diagnosis and foreign body extraction from airways are essential. We performed a retrospective study on rigid bronchoscopy outcomes due to suspected foreign body aspiration. A total of 66 children were admitted to the Chair and Department of Pediatric Otolaryngology, Phoniatrics and Audiology, Medical University of Lublin between 2015 and 2020 and underwent rigid bronchoscopy in general anesthesia due to suspected foreign body aspiration. We analyzed the data, including patients age and sex, reported complaints, and bronchoscopy findings. Analyzed children were aged from 8 months to 17 years old; 74.24% of them were under 3 years old during the procedure, and most of the operated patients were males. In 36.36% cases, no foreign body was identified, and 57.14% foreign bodies were located in right main bronchus. A total of 80.95% of foreign bodies extracted from airways were organic, mostly nuts. Diagnosis and treatment of suspected foreign body aspiration requires consistent cooperation between pediatricians, pulmonologists, anesthesiologists, and otolaryngologists.

## 1. Introduction

Foreign body aspiration in children is a common condition, which may lead to serious complications; it is estimated that it may contribute to 7% of deaths related to trauma in children aged between 1 to 3 years old, reaching almost 40% in infants posing as the most frequent cause of death in this age range [1,2,3,4].

Anatomical and physiological actors contributing to high risk of foreign body aspiration include lack of molar teeth, high larynx position, dysfunctional epiglottis, undeveloped chewing and swallowing reflex, and uncoordinated swallowing and breathing [3,4,5]. Additional risk factors in small children are excessive motion activity and exploration via digestive system; insufficient guardianship; access to inappropriate toys with small elements; and inappropriate food intake, for instance, chocolate bars with nut fragments [6,7].

Foreign body aspiration may lead to obstruction and numerous long-term consequences such as recurrent lung infections, atelectasis, and bronchiectasis [8]. A foreign body remaining in the airways for a long period of time may manifest as pneumonia or asthma, which extends the process of diagnosis and application of adequate treatment [9,10]. 

In order to prevent complications, rapid diagnosis and immediate foreign body extraction is recommended. Rigid bronchoscopy in general anesthesia is the procedure of choice; it allows for secure ventilation and suitable tool usage for efficient foreign body extraction [11,12].

Foreign body aspiration symptoms are not specific; in some cases, they can be absent, and they are also symptoms that can be seen in other lung diseases. Signs of foreign body aspiration include cough, dyspnea, and choking. However, no single symptom, medical interview element, or diagnostic examination is specific nor sensitive enough to unequivocally indicate aspiration identification; only bronchoscopy provides certainty [4,13].

Even in questionable cases, bronchoscopy provides a reliable diagnostic and therapeutic tool [1,8]. We have to remember that foreign body aspiration is an emergency, and in potentially stable cases, the patient’s condition may drastically worsen, which is why fast and proper diagnosis should be stated and adequate therapeutic protocol should be applied [14].

## 2. Materials and Methods

Retrospective medical records analysis of 66 patients admitted to Chair and Department of Pediatric Otolaryngology, Phoniatrics and Audiology, Medical University of Lublin between 2015 and 2020 who underwent rigid bronchoscopy in general anesthesia due to suspected foreign body aspiration was performed. We excluded cases in which medical history was incomplete. All of children were examined by an experienced pediatrician and otolaryngologist, chest X-ray was performed, and the patients’ guardians signed conscious and informed consent forms for rigid bronchoscopy under general anesthesia. We analyzed the following features: patients age and sex, reported complaints, and bronchoscopy findings. 

Medical records showed that general condition of most of the patients allowed for the application of 6 h fasting before procedure; in 5 cases, bronchoscopy was performed urgently, ignoring fasting interval. Every procedure took place in an operation ward. Size and length of bronchoscopy tools was adjusted in regard to patients age and weight. After identification of the foreign body in airways and assessment of its location, extraction was performed using forceps. After foreign body or/and mucus removal, a second look was performed in order to evaluate mucosa condition and identify any remaining fragments of foreign body or iatrogenic injury.

In 2 cases, the foreign body could not be extracted in rigid bronchoscopy as airway perforation was suspected (fragment of turkey bone and metal office pins); children were transported to a referential medical facility and underwent a procedure conducted by a thoracic surgeon.

Data were collected in Microsoft Excel database and analyzed in Statistica software. Qualitative data were presented as percentages, and quantitative data as averages. Difference between values was assessed with Student’s *t*-test. Statistical relevance level was recognized for *p* < 0.05.

## 3. Results

**Figure 1:**
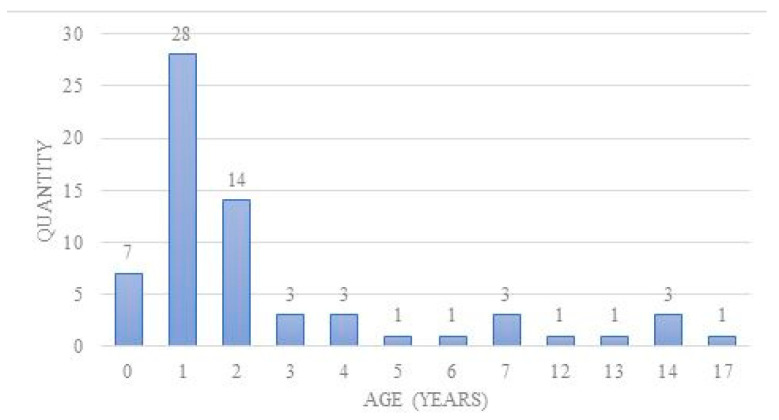
Age of patients suspected of foreign body aspiration.

**Figure 2:**
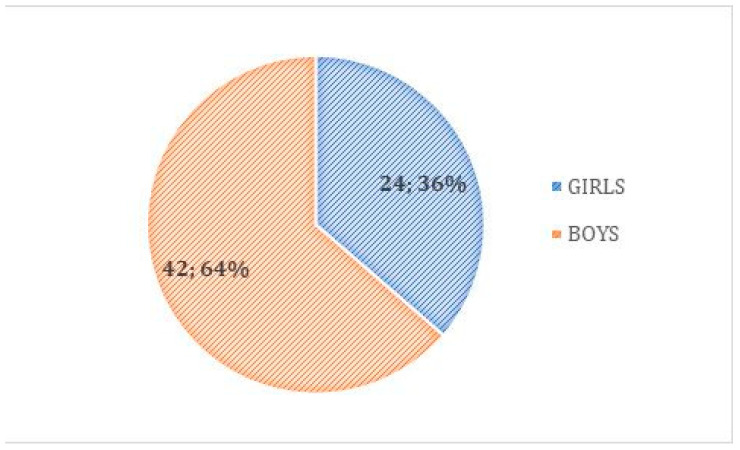
Sex of patients suspected of foreign body aspiration.

Analyzed children were aged from 8 months to 17 years old; average age was 2.92 years old. Quantity of each age group is presented on the graph below (Figure 1). Among the operated patients, 49 (74.24%) were no older than 3 years of age at the time of the procedure. Most of the children suspected of foreign body aspiration were boys, at 42 cases (63.64%); girls were operated upon in 24 cases (36.36%) (Figure 2).

Most raised complaints included choking (54 cases; 81.81%), swallowing foreign object (6 cases; 9.09%), cough (3 cases; 4.55%), dyspnea (2 cases; 3.03%), and dyspepsia (1 case; 1.52%).

**Figure 3:**
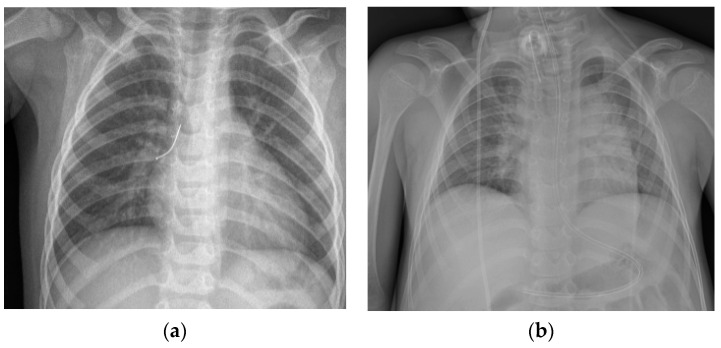

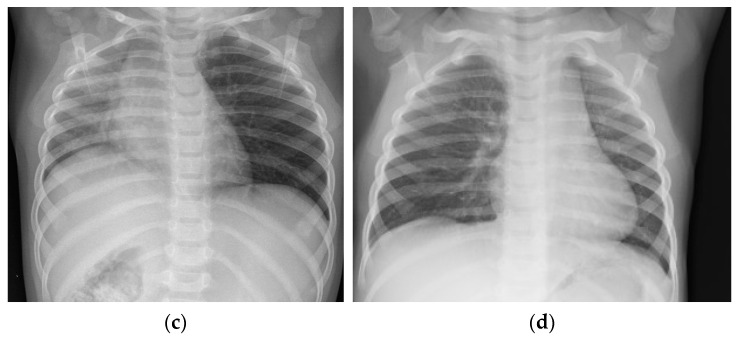
X-ray imaging results. (**a**) Evidence of foreign body in right bronchi; (**b**) intensified vascular pattern; (**c**) mediastinum dislocation to right side; (**d**) mediastinum dislocation to left side.

In the analyzed group, X-ray imaging results included: evidence of foreign body (metal objects), unilateral pneumothorax or atelectasis, intensified vascular pattern, emphysema, and mediastinum dislocation (Figure 3).

In 24 cases (36.36%), no foreign body was identified during rigid bronchoscopy, while in the remaining 42 cases (63.64%), foreign body was present in airways. Foreign bodies were located in the right main bronchus in 20 cases (57.14%) and left bronchus in 18 cases (42.86%). In 2 cases (4.76%), aspirated material (barium, popcorn) was present in both bronchi, and in 2 cases (4.76%), in the trachea (medical needle, unidentified organic foreign body).

Extracted foreign bodies were organic in 34 cases (80.95%), while 8 of them (19.05%) were inorganic. Characteristics of aspirated foreign objects are presented in the table below (Table 1). The most common organic foreign bodies included all kinds of nuts (24 cases; 57.14%).

Average age of patients who aspirated organic foreign bodies was 2.44 years old, while average age of patients who aspirated inorganic foreign bodies was 4.88 years old. Observed disparity was not statistically significant (*p* > 0.05). 

Inflammation of lower airways was observed during rigid bronchoscopy in 19 cases (27.79%).

## 4. Discussion

In the analyzed studies, the average age of patients suspected of foreign body aspiration who underwent rigid bronchoscopy was 3 years old, while most of them (52–81%) did not exceed 3 years old [1,3,9,15,16]. It is believed that the third year of life carries the greatest risk of foreign body aspiration. Our study confirmed this statistical data.

Recent studies unequivocally indicate that among patients qualified for bronchoscopy with suspected foreign body aspiration, most cases concern boys (59–64%) [9,11,15]. Male predisposition may result from more excessive physical activity in preschool developmental period compared to girls. Data collected in our retrospective analysis are compatible with the aforementioned statistics.

The most frequently observed symptoms suggesting foreign body aspiration are cough (94–96%), choking (71–83%), and dyspnea (44–61%) [6,9,11]. In a study conducted in Turkey, choking episodes observed by guardians was reported in 91% of cases of positive bronchoscopies (foreign body in airways was present) and 54% of cases of negative bronchoscopies (foreign body in airways was absent) [1]. It is believed that credible episode of choking appears as the most specific and sensitive prognostic factor of foreign body identification in bronchoscopy; its specificity is estimated at 46%, while sensitivity reaches 91% [1,16]. Longstanding clinical experience unequivocally indicates that bronchoscopy is necessary in cases of reliable medical interview with observed choking episodes, even if physical examination and imaging investigations are questionable.

Chest X-ray is a routine imaging investigation when foreign body aspiration is suspected, although its value in deriving infection from foreign body aspiration is minor and it must be remembered that a normal radiograph does not rule out the presence of a foreign body in the airways. It is estimated that unchanged chest X-ray image frequency, despite foreign body presence, could reach up to 66% of patients [1,5,8,17,18].

When physical examination reveals dyspnea, cough, and excessive respiratory effort or fever, and chest X-ray image presumes a plausible foreign body aspiration, even if no choking episode was observed, it is recommended that rigid bronchoscopy be performed. Oversight and foreign body retention in lower airways may lead to serious adverse results [2,19]. Chest X-ray may show obvious evidence of foreign body (metal objects), but most times, radiologic image indirectly suggests the presence of an obstacle in the lower airways; in these cases, unilateral pneumothorax or atelectasis, emphysema, and mediastinum dislocation could be observed [4,6].

Bilateral radiological changes have lesser diagnostic value compared to unilateral ones, but this does not exclude the possibility of foreign body aspiration. In one of the analyzed cases, a 14-year-old girl with cerebral palsy experienced a choking episode during an investigation of the passage of barium sulphate contrast medium through the gastrointestinal tract, which resulted in barium aspiration to the airways and required two bronchoscopies in a two-day interval in order to remove the remaining contrast medium.

Suspected foreign body aspiration requires performing rigid bronchoscopy in less than 24 h from a choking episode [20].

Absence of foreign body in airways during bronchoscopy is observed with varied incidence, depending on medical facility, between 10% and 75% of cases [1,3,4,15,21]. Percentage of negative bronchoscopies in the analyzed group stood at 36.36% and fit into the aforementioned range.

Recent studies suggest that foreign bodies can be located in the right main bronchus in 30–60% of cases, left main bronchus in 25–60% of cases, both bronchi in 0–5% of cases, and in the trachea in 1.5–34% of cases [1,2,3,22,23]. In analyzed cases, foreign bodies were mostly extracted from the right main bronchus (20 cases; 57.14%). More frequent localization of aspirated objects in right main bronchus could be related to greater diameter and more straight structure compared to left main bronchi [4,6,16]. 

Studies suggest that most of the foreign bodies extracted from children’s airways are organic [1,4,9]. Results obtained from our study confirm this statement—in 34 cases (80.95%), foreign bodies observed in bronchoscopy were organic, while only 8 patients (19.05%) were aspirated objects inorganic. It is reported that among organic foreign bodies extracted from airways, the most frequent are nuts, meat, vegetables, and fruits [2,3,4,5,14,16]; in our study, nuts were found most commonly (24 cases; 57.14%). Inorganic foreign bodies are seen in 7–25% positive bronchoscopies; the most common inorganic objects include fragments of pens and small plastic toys [1,9]. Studies suggest that organic foreign bodies are mainly aspirated by preschool children, while inorganic objects are more commonly extracted from older children [5,20]. A similar tendency was observed in our study.

A study conducted in Turkey showed that in 6% of cases, inflammation of mucosa was observed during bronchoscopy [1]. The longer delay of bronchoscopy, the greater incidence and intensity of an inflammatory condition in the lower airways. Children who underwent a procedure after 24 h from foreign body aspiration can experience bronchitis in up to 34% of cases [9]. Our study revealed an inflammation ratio in almost 28% of cases.

Rigid bronchoscopy in all patients hospitalized in the Chair and Department of Pediatric Otolaryngology, Phoniatrics and Audiology, Medical University of Lublin was performed as soon as possible. Late admittance to the Emergency Ward after possible foreign body aspiration undoubtfully had an impact on delayed procedure performance and most likely bronchitis development.

## 5. Conclusions

The study results confirmed that children who are most likely to experience foreign body aspiration are boys under 3 years of age, and the most common kind of foreign body is organic matter.

Diagnosis and treatment of children with suspected foreign body aspiration are complex processes and require close cooperation between pediatricians, anesthesiologists, and otolaryngologists. Rigid bronchoscopy in general anesthesia plays a basic role in therapeutic algorithm.

It is important to educate children’s guardians as to how to prevent foreign body aspiration and explain what consequences can it cause. Rapid bronchoscopy and foreign body extraction may minimalize risk of long-term complications.

## Figures and Tables

**Table 1:** Kind and quantity of foreign bodies aspirated to lower airways.

Kind of Foreign Body	Type	Quantity
Organic	Nuts	24
	Undefined organic foreign body	3
	Apple	2
	Popcorn	2
	Cauliflower	1
	Carrot	1
	Turkey bone	1
Inorganic	Medical needle	1
	Office pin	1
	Expanding foam	1
	Metal screw	1
	Barium	1
	Sponge	1
	Food wrap	1
	Pen aglet	1

## Data Availability

Raw data were generated at Medical University of Lublin. Derived data supporting the findings of this study are available from the corresponding author A.P. on request.
